# GPX8 as a potential prognostic marker in gastric and colorectal cancer

**DOI:** 10.1097/MD.0000000000039815

**Published:** 2025-01-17

**Authors:** Ya-Wen Zou, Yun Cheng, Zhi-Lin Liu, Hai-Tao Wang

**Affiliations:** a Department of Gastrointestinal Surgery, Third Affiliated Hospital of Soochow University, Changzhou, China; b Department of Gastrointestinal Surgery, Changzhou Geriatric Hospital Affiliated to Soochow University, Changzhou, China.

**Keywords:** colorectal cancer, GPX8, lymphocyte infiltration, prognosis, stomach adenocarcinoma

## Abstract

Gastric and colorectal cancers are common malignancies with high incidence and mortality worldwide. Early detection and individualized treatment are crucial to improving patient outcomes. Glutathione peroxidase-8 (GPX8), a member of the glutathione peroxidase family, emerges as a potential target for intervention in the treatment of various cancers. This study investigated the potential of GPX8 as a prognostic marker in patients with gastric and colorectal adenocarcinoma. The study employed a multi-omics approach to analyze GPX8 expression in both tumor and adjacent normal tissue of stomach adenocarcinoma (STAD), colon adenocarcinoma (COAD) and rectum adenocarcinoma patients. The Cancer Genome Atlas database was used to download the microarray data of GPX8 and clinical information for cancer patients. The TIMER database and TNMplot database were used to systematically evaluate the association of GPX8 with tumor-infiltrating lymphocytes in adenocarcinoma. Immunohistochemistry is used to detect GPX8 expression in clinical tumors and adjacent normal tissues. Univariate Cox analysis was performed to explore the relationship between GPX8 expression, immune cell levels, and the prognosis in cancer patients. GPX8 was significantly upregulated in tumor tissue and was associated with a poor prognosis in STAD and COAD patients. Furthermore, high GPX8 expression was found to be correlated with a higher degree of CD4+ T cell-infiltrating in COAD and neutrophil-infiltrating in STAD, indicating that GPX8 may play a role in immune evasion in cancer progression. This study highlights the potential of GPX8 as a prognostic marker in STAD and COAD, providing valuable insight into the development of personalized treatment strategies for cancer patients.

## 1. Introduction

Adenocarcinoma is a malignant tumor and a subtype of cancer that is classified into 2 main categories: carcinoma and sarcoma. Stomach adenocarcinoma (STAD) is a type of malignancy that arises within the stomach and presents a significant threat not only to the digestive system but also to other organs and systems through metastasis. According to the latest GLOBOCAN 2020 report, gastric cancer ranks fifth as the most common cancer type and 4th as the leading cause of death from cancer globally. Of note, STAD accounts for 5.6% of all new cancer cases and 7.7% of cancer-related deaths worldwide.^[1]^ While the incidence and mortality rates of gastric cancer have declined in some regions, such as the United States and Western Europe, the burden remains high in others, such as Northeast Asia, Eastern Europe, and Latin America.^[2]^ Colon adenocarcinoma (COAD), on the other hand, is a major cause of cancer mortality that affects both men and women almost equally.^[3,4]^ Both STAD and COAD have high rates of cancers and mortality in China, highlighting the need to develop novel therapeutic interventions for these diseases.^[5]^

The glutathione peroxidase (GPX) family of antioxidant enzymes is a crucial group of selenoenzymes in mammals that play a vital role in mitigating the harmful effects of H_2_O_2_ or hydroperoxides.^[6]^ Among its members, glutathione peroxidase-8 (GPX8) stands out as a non-selenium congener with glutathione peroxidase activity.^[7]^ This endoplasmic reticulum protein has been linked to hydrogen peroxide production, endoplasmic reticulum stress, apoptosis, and the regulation of calcium homeostasis and signaling.^[8]^ While the function of GPX8 in cancer remains largely unexplored, studies have shown that it is involved in various physiological processes and tumorigenesis, and has been associated with poor prognostic outcomes in gastric, breast, and lung cancers.^[9–11]^ However, there is a paucity of research on the relationship between GPX8 and COAD.^[12]^

This study explored the expression of GPX8 in patients with STAD and colorectal adenocarcinoma, and its relevance to patient prognosis, utilizing clinical sample analysis, bioinformatics databases, and statistical methods. Furthermore, the association between GPX8 and tumor-infiltrating lymphocytes in the tumor microenvironment, as well as the biological functions of GPX8 were also investigated, providing valuable insights into personalized treatment strategies for cancer patients.

## 2. Methods

### 2.1. TIMER database

The TIMER2.0 platform (http://timer.cistrome.org/)^[13]^ is a comprehensive web server solution that provides advanced analysis and visualization capabilities for tumor infiltrating immune cells. It offers an effective approach to estimating immune infiltration levels for data from The Cancer Genome Atlas (TCGA). This is achieved by utilizing 6 state-of-the-art algorithms rather than a single algorithm. Additionally, TIMER2.0 comprises 4 modules aimed at analyzing the relationship between immune infiltration and clinical or genetic characteristics, and 4 modules intended for discovering cancer-related associations in the TCGA cohorts. In the present study, we used the TIMER database to identify differences in GPX8 expression between STAD, COAD, and adjacent normal tissues. The relationship between GPX8 expression and immune cells was also evaluated by the TIMER database.

### 2.2. TNMplot database

The TNMplot database (www.tnmplot.com)^[14]^ includes 56,938 unique samples collected from gene expression omnibus, genotypic tissue expression, TCGA, and Therapeutically Applicable Research To Generate Effective Treatments. It is an integrated database using available transcriptome-level datasets by comparing normal, tumor and metastatic data across most genes. The TNMplot database was utilized to identify differences in GPX8 expression between STAD, COAD, rectum adenocarcinoma (READ) and adjacent normal tissues.

### 2.3. TCGA database

TCGA (https://portal.gdc.cancer.gov) molecularly characterized over 20,000 primary cancers and matched normal samples spanning 33 cancer types. Over the next dozen years, TCGA generated more than 2.5 petabytes of data, which has already led to improvements in our ability to diagnose, treat, and prevent cancer. In the present study, we utilized the TCGA database to evaluate the prognostic role of GPX8 in STAD (n = 415), COAD (n = 166), and READ (n = 458) patients.

### 2.4. Survival analysis

KM curve and Cox regression were used for survival analysis. The cancer tissues were divided into high and low expression groups according to the median of gene expression. A Cox regression analysis was performed to adjust for age and sex.

### 2.5. Sample selection and immunohistochemical staining

A total of 100 cancer patients were enrolled and followed up, of which 50 patients were diagnosed with STAD by pathology and the rest with colorectal adenocarcinoma. Paraffin tissue specimens of surgically resected tissue were selected for this study, and surgical margin tissue was used as the control. All cases had complete clinical data and had not received chemotherapy, radiotherapy, or other antitumor adjuvant therapy before surgery. The study was conducted in accordance with the Declaration of Helsinki and approved by the Ethics Committee of the Third Affiliated Hospital of Soochow University. Written informed consent was obtained from each participant.

To prepare the tissue for sectioning, first the tissue was sectioned into 5-μm thickness and subjected to immunohistochemical staining. Following deparaffinization, endogenous peroxidases were blocked using 3% H_2_O_2_ for 20 minutes. Antigen retrieval was achieved by heating the samples in 10 mmol/L citrate buffer (pH 6.0) at 95 °C for 60 minutes. The sections were then incubated with an anti-GPX8 antibody (Abcam, Cambridge, MA) at a dilution of 1:300 at 4 °C overnight. Immunohistochemical staining was graded visually based on the intensity of stained cells, categorized as low (+), moderate (++), or high (+++). Immunostaining was evaluated by 2 independent pathologists. Three positions were selected for each tissue slice, and the average optical density values were determined. The assessment of immunostaining involved the inspection of 2 pathologists. Each tissue slice was reviewed at 3 distinct locations, and the corresponding optical density values were measured for each. Subsequently, the average of the 3 optical density values was computed and employed as an indicator for the GPX8 expression measurement.

### 2.6. Statistical analyses

In the statistical analyses, the data are displayed as the mean value with standard deviation. Statistical significance is denoted by a *P*-value <.05. Analysis was conducted using SAS 9.4 software.

## 3. Results

### 3.1. Expression of the GPX8 in pan-cancer perspective

**Figure 1.**
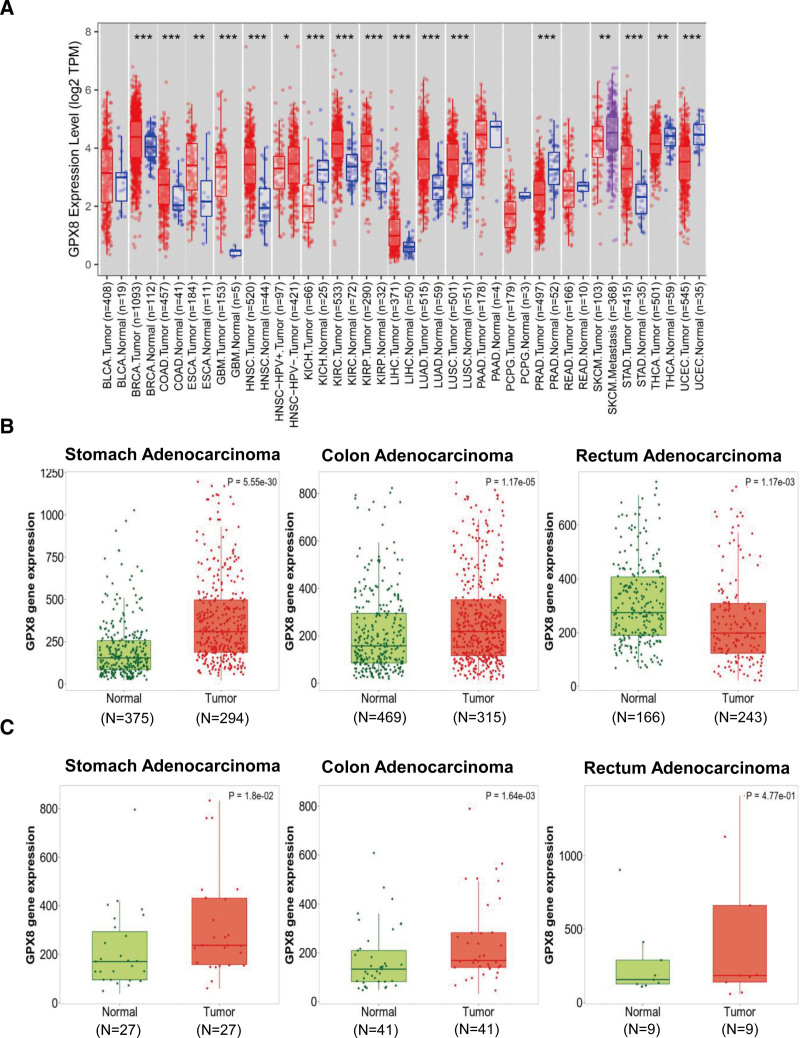
Expression pattern of GPX8 in pan-cancer perspective. (A) Expression pattern of GPX8 in pan-cancer perspective (*P* ≥ .05; *P < .05; **P < .01; ***P < .001) in TIMER database. (B and C) GPX8 expression in paired and unpaired STAD, COAD, and READ tumor tissues and normal tissues (TNMplot database). COAD = colon adenocarcinoma; GPX8 = glutathione peroxidase-8; READ = rectum adenocarcinoma; STAD = stomach adenocarcinoma.

To clarify the function of GPX8 in cancer, we explored GPX8 expression in 21 different cancers in a pan-cancer analysis. As shown in Figure 1A, GPX8 was significantly differentially expressed in 17 out of 21 cancer tissues. Specifically, GPX8 was markedly overexpressed in BRCA, COAD, ESCA, GBM, HNSC, KIRC, KIRP, LIHC, LUAD, LUSC, and STAD tumor tissues compared with normal tissues, while it was downregulated in KICH, PRAD, THCA, and UCEC tumor tissues (*P* < .01). Moreover, compared with HNSC-HPV- and SKCM, the expression of GPX8 in HNSC-HPV+ and SKCM. Metastasis tissues are significantly down-regulated and up-regulated (*P* < .05), respectively. These findings indicate differential expression of GPX8 across various cancer types and significant differences in expression levels between tumor and normal tissues.

Subsequently, GPX8 expression was further explored in STAD, colorectal adenocarcinoma, and paracancer tissues in the TNMplot database. The results showed that the expression level of GPX8 was significantly higher in STAD and colorectal adenocarcinoma tumor tissues compared to normal tissues, but significantly lower in READ (*P* < .01, Fig. 1B). While comparing paired tumor and normal gene array data, a significantly higher expression of GPX8 in STAD and COAD was observed (*P* < .05), with no significant difference in GPX8 expression in READ (Fig. 1C). These results above indicated that the expression of GPX8 is higher in STAD and COAD compared to normal tissues.

### 3.2. GPX8 is closely related to the poor prognosis of STAD, COAD, and READ patients

**Figure 2.**
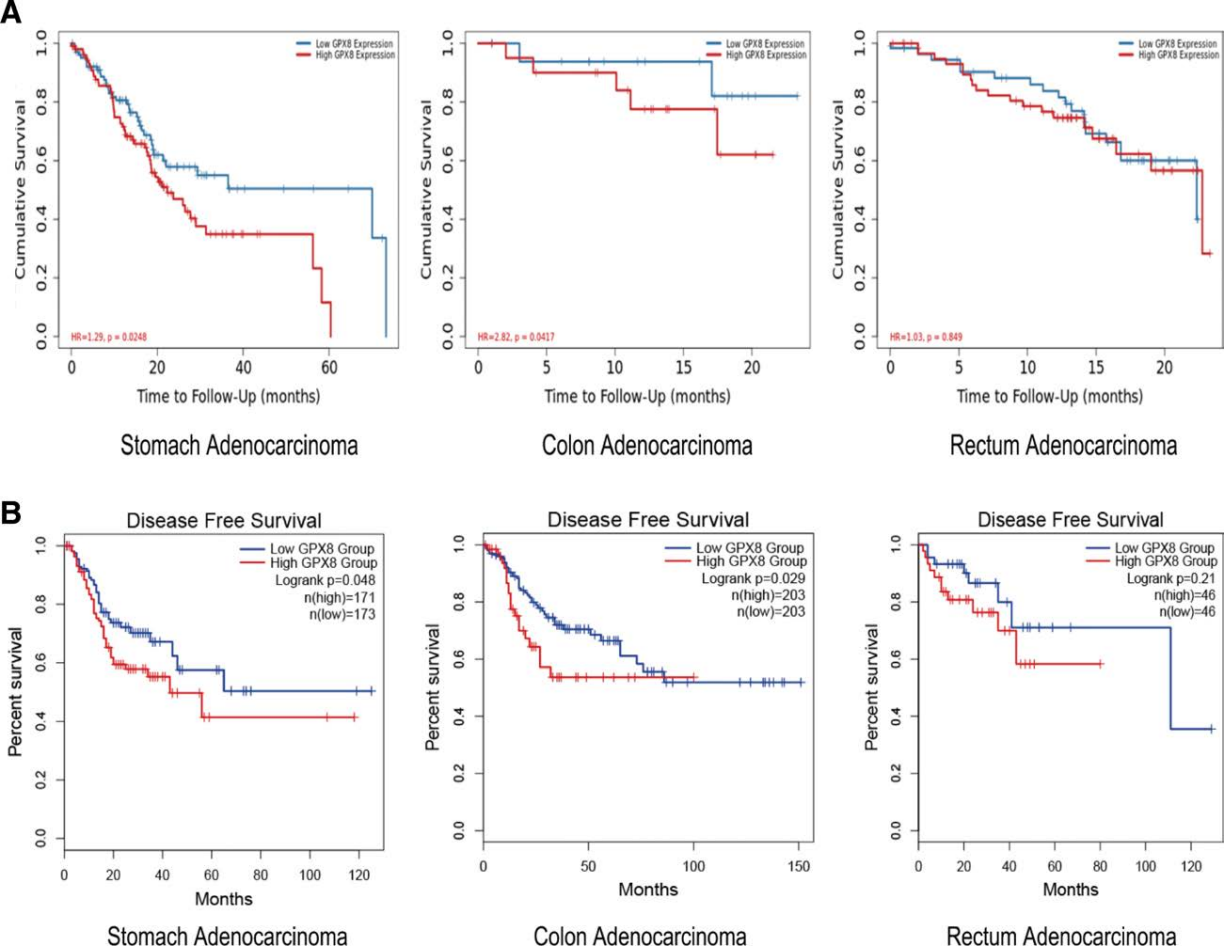
GPX8 is closely related to the poor prognosis of STAD and colorectal adenocarcinoma. Kaplan–Meier survival curve was used to analyze the relationship between GPX8 and OS (A) or DFS (B) of STAD, COAD and READ patients. COAD = colon adenocarcinoma; DFS = disease-free survival; GPX8 = glutathione peroxidase-8; READ = rectum adenocarcinoma; STAD = stomach adenocarcinoma.

Subsequently, we explored the correlation between GPX8 expression and independent prognostic factors, including overall survival (OS) and disease-free survival (DFS) of the patients. High expression of GPX8 has proved to be significantly associated with reduced OS and DFS in patients with STAD (HR = 1.29, *P* = .0248) and COAD (HR = 2.82, *P* = .0417) (Fig. 2A/B). Consistently, the results in Table 1 revealed that STAD patients from the TCGA database with high GPX8 expression exhibited lower OS (HR = 1.268 [1.083–1.484], *P* = .003) and DFS rates (HR = 1.513 [1.015–2.011], *P* = .048) than those with a low GPX8 expression. Similarly, patients with COAD and high GPX8 expression also displayed reduced OS (HR = 1.647 [1.094–2.478], *P* = .017) and DFS rates (HR = 1.795 [1.352–2.238], *P* = .031) compared to individuals with low expression of GPX8.

**Table 1:** Cox regression analysis for the correlation between GPX8 expression and independent prognostic factors in STAD, COAD, and READ.

Cancer	Hazard ratio	Standard error	95% confidence interval	Z	*P*
OS					
STAD	1.268	0.081	1.083–1.484	2.946	.003
COAD	1.647	0.209	1.094–2.478	2.391	.017
READ	1.144	0.109	0.924–1.415	1.234	.217
DFS					
STAD	1.513	0.254	1.015–2.011	1.942	.048
COAD	1.795	0.226	1.352–2.238	2.154	.031
READ	2.014	0.524	0.987–3.041	1.183	.226

All the hazard ratio were adjusted by age and gender.

COAD = colon adenocarcinoma; DFS = disease-free survival; OS = overall survival, READ = rectum adenocarcinoma, STAD = stomach adenocarcinoma.

### 3.3. The correlation of GPX8 and tumor-infiltrating lymphocytes in STAD and COAD

The Spearman correlation coefficients were performed to quantitatively evaluate the correlation between GPX8 and 21 different immune cells. Our findings indicated a significant correlation between various immune cells and GPX8 in STAD and COAD (Table 2). Specifically, cancer-associated fibroblast, common myeloid progenitor, endothelial cell, eosinophil, granulocyte–monocyte progenitor, hematopoietic stem cell, macrophage, mast cell, monocyte, myeloid dendritic cell, neutrophil, and NK cell were positively correlated with GPX8 expression in both STAD and COAD. While B cell, common lymphoid progenitor, myeloid derived suppressor cells, plasmacytoid dendritic cell, T follicular helper cell, T cell NK were negatively correlated. Interestingly, CD8+ T cells demonstrated a positive correlation with GPX8 expression in STAD, but not in COAD. Conversely, CD4+ T cell and regulatory T cell exhibited a positive correlation with GPX8 expression in COAD but not in STAD.

**Table 2:** Spearman correlation coefficient between GPX8 expression and immune cell infiltration in STAD and COAD.

Infiltrates	STAD			COAD		
	Correlation coefficient (Spearman)	*P*	FDR	Correlation coefficient (Spearman)	*P*	FDR
B cell	‐0.243	<.001	<0.001	‐0.230	<.001	<0.001
Cancer associated fibroblast	0.499	<.001	<0.001	0.573	<.001	<0.001
Common lymphoid progenitor	‐0.238	<.001	<0.001	‐0.206	<.001	<0.001
Common myeloid progenitor	0.146	.004	0.015	0.057	.347	0.507
Endothelial cell	0.308	<.001	<0.001	0.460	<.001	<0.001
Eosinophil	0.125	.015	0.043	0.010	.874	0.920
Granulocyte-monocyte progenitor	0.233	<.001	<0.001	0.314	<.001	<0.001
Hematopoietic stem cell	0.450	<.001	<0.001	0.492	<.001	<0.001
Macrophage	0.627	<.001	<0.001	0.411	<.001	<0.001
Mast cell activated_CIBERSORT	0.232	<.001	<0.001	0.236	<.001	<0.001
Myeloid derived suppressor cells	‐0.177	.001	0.002	‐0.124	.013	0.037
Monocyte	0.234	<.001	<0.001	0.369	<.001	<0.001
Myeloid dendritic cell	0.296	<.001	<0.001	0.495	<.001	<0.001
Neutrophil	0.285	<.001	<0.001	0.409	<.001	<0.001
NK cell	0.129	.012	0.035	0.256	<.001	<0.001
Plasmacytoid dendritic cell	‐0.164	.001	0.005	‐0.269	<.001	<0.001
T cell CD4+	‐0.448	<.001	<0.001	0.303	<.001	<0.001
T cell CD8+	0.389	<.001	<0.001	‐0.213	<.001	0.002
T cell follicular helper	‐0.293	<.001	<0.001	‐0.227	<.001	<0.001
T cell NK	‐0.156	.002	0.008	‐0.165	.006	0.020
T cell regulatory (Tregs)	‐.192	<.001	<0.001	0.374	.001	<.001

All the Spearman correlation coefficients were adjusted by purity.

COAD = colon adenocarcinoma, FDR = false discovery rate, READ = rectum adenocarcinoma, STAD = stomach adenocarcinoma.

**Figure 3.**
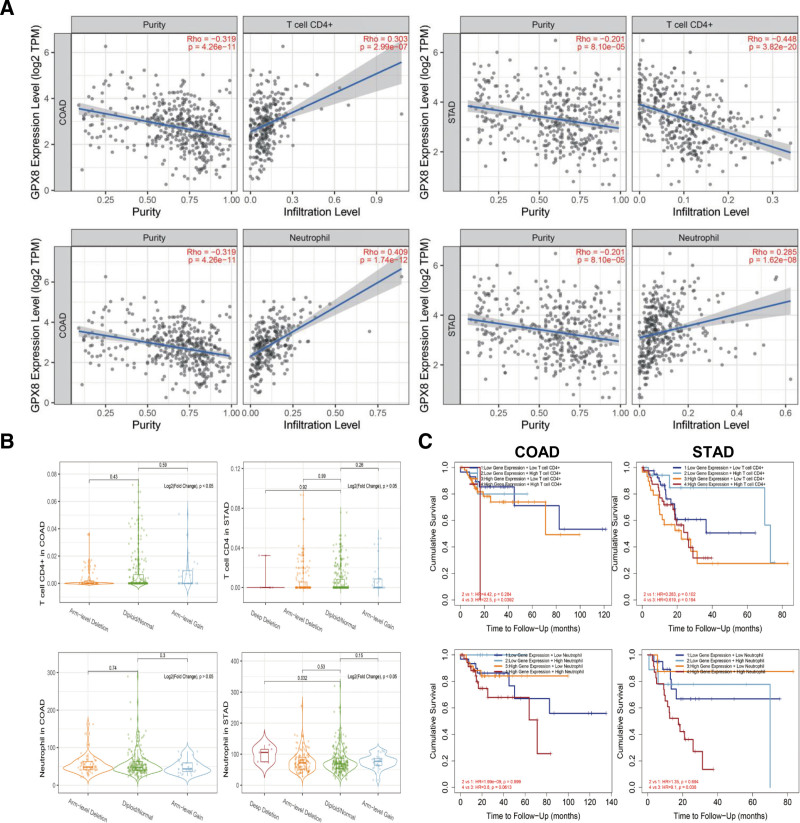
The association of GPX8 expression and tumor infiltrating lymphocytes (CD4+ T cell and neutrophil) in STAD and COAD. (A) Scatter of immune infiltrating lymphocytes and GPX8 expression level in STAD and COAD. (B) Comparing immune infiltration distribution by the sCNA status in STAD and COAD. The gene level, including “deep deletion,” “arm-level deletion,” “diploid/normal,” “arm-level gain,” and “high amplification,” was defined by GISTIC2.0. (C) Survival curves of OS between GPX8-high or -low and immune infiltrating-high or -low patients with STAD and COAD. COAD = colon adenocarcinoma; GPX8 = glutathione peroxidase-8; OS = overall survival; STAD = stomach adenocarcinoma.

We further investigated the association between immune infiltrates and somatic copy number variation (CNV). Among the 21 immune cells analyzed, CD4+ T cells and neutrophils consistently exhibited a stable association with high expression of GPX8 (Fig. 3A), the infiltration level of CD4+ T cell was positively correlated with GPX8 expression in COAD (Rho = 0.303, *P* = 2.99e‐07) but negatively correlated in STAD (Rho = −0.448, *P* = 3.82e‐20), while the infiltration level of neutrophils was positively correlated with GPX8 expression in both COAD (Rho = 0.409, *P* = 1.74e‐12) and STAD (Rho = 0.285, *P* = 1.62e‐08). When analyzing the association between immune infiltrates and somatic CNV, we found that CD4+ T cell infiltration level was associated with somatic CNV in COAD, and neutrophil infiltration level was associated with somatic CNV in STAD (Fig. 3B). Furthermore, our results revealed that GPX8-high patients with high CD4+ T cell infiltration in COAD exhibited a shorter survival time compared to those with low CD4+ T cell infiltration (*P* = .0392), which was similar to GPX8-high patients in STAD with high neutrophil infiltration compared to those with low neutrophil infiltration (*P* = .036) (Fig. 3C). Additionally, the results of Cox regression analysis (Table 3) demonstrated that patients with low CD4+ T cell infiltration levels had higher OS rates (HR = 10.554 [1.204–92.636], *P* = .033) compared to those with high infiltration levels in COAD, while patients with low neutrophil infiltration levels had higher overall survival rates (HR = 11.348 [3.386–38.028], *P* = .041) compared to those with high infiltration levels in STAD.

**Table 3:** Cox regression analysis for the correlation betweenGPX8 expressions and immune infiltration level in STAD and COAD.

	Hazard ratio	Standard error	95% confidence interval	Z	*P*
STAD					
T cell CD4+	4.959	2.046	0.090–273.327	0.783	.434
Neutrophil	11.348	0.617	3.386–38.028	1.970	.041
COAD					
T cell CD4+	10.554	1.108	1.204–92.636	2.126	.033
Neutrophil	3.998	0.792	0.847–18.884	1.260	.237

All the hazard ratio were adjusted by age, gender and purity.

COAD = colon adenocarcinoma, STAD = stomach adenocarcinoma.

### 3.4. Expression and prognosis analysis of GPX8 in STAD and COAD

**Figure 4.**
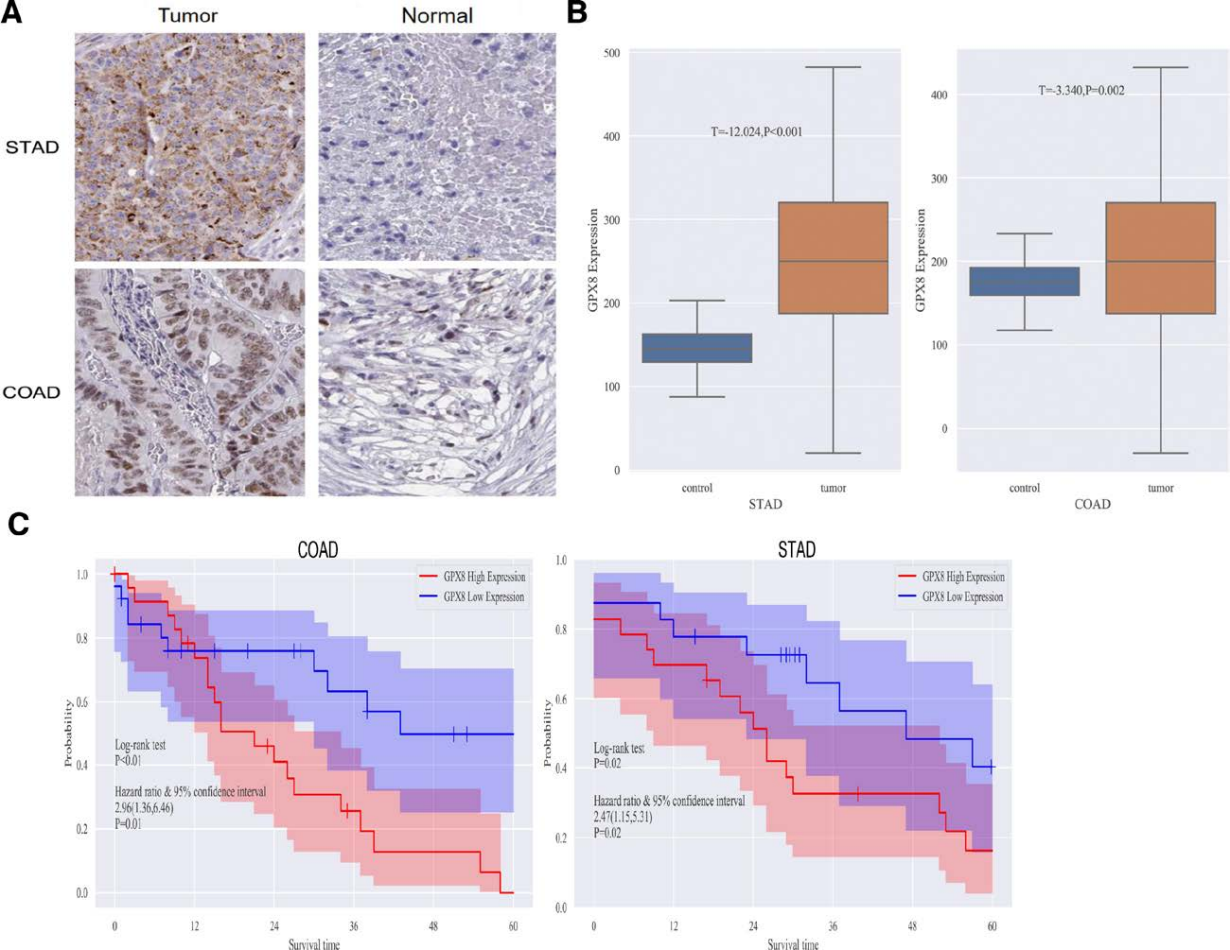
Expression and prognosis analysis of GPX8 in STAD and COAD. (A) Immunohistochemical images of tumor and adjacent normal tissues from representative patients with STAD and COAD. (B) The expression of GPX8 in tumor is higher than that in normal tissues in STAD and COAD. (C) The Kaplan–Meier curve showed that a low GPX8 expression was associated with higher OS in STAD patients. CI = confident interval; COAD = colon adenocarcinoma; GPX8 = glutathione peroxidase-8; HR = hazard ratio; OS = overall survival; STAD = stomach adenocarcinoma.

Immunohistochemistry was conducted to assess GPX8 expression levels in tumor tissues and adjacent normal tissues from 50 patients with STAD and 50 patients with COAD. The immunohistochemical results for tumor and normal tissues from representative patients are displayed in Figure 4A. As shown in Figure 4B, GPX8 expression was significantly higher in tumor tissues compared to adjacent normal tissues in both STAD (*t* = ‐12.024, *P* < .001) and COAD (*t* = ‐3.340, *P* = .002). Based on the median GPX8 expression level in the tumor tissues, we categorized the patients into 2 groups, a high GPX8 expression group and a low GPX8 expression group. Furthermore, as depicted in Figure 4C, the OS rate of patients with high GPX8 expression was significantly lower than that of the low GPX8 expression in both STAD (*P* < .01, HR [95%CI] = 2.96 (1.36,6.46)) and COAD (*P* < .01, HR [95%CI] = 2.38[1.37,4.13]). Our study supports the findings from previous studies based on public databases, suggesting that GPX8 may serve as a potential biomarker for prognostic evaluation in patients with STAD and COAD.

## 4. Discussion

This study aimed to investigate the correlation between GPX8 and COAD and STAD. Our analysis revealed a significantly higher GPX8 expression in tumor tissues compared to adjacent normal tissue in both STAD and COAD, and high expression was associated with reduced OS. We also found a significant correlation between various immune cells and high expression of GPX8 in both STAD and COAD. CD4+ T cell and neutrophils exhibited a stable association with high expression of GPX8. Immunohistochemical analysis of tissue samples obtained from our hospital confirmed the relationship between GPX8 and STAD and COAD. Patients with high GPX8 expression had significantly lower overall survival rates compared to those with low GPX8 expression in both STAD and COAD. Overall, our study supports the potential of GPX8 as a biomarker for prognostic evaluation in patients with STAD or COAD.

Previous studies have investigated the potential association between GPX8 expression and prognosis in patients with COAD and STAD. Some studies have reported that high GPX8 expression is correlated with poor prognosis in COAD and STAD patients. For instance, Ren et al^[15]^ reported that high GPX8 expression was associated with worse overall survival and disease-free survival in COAD patients, while Zhang et al^[16]^ found that high GPX8 expression was correlated with unfavorable survival outcomes in STAD patients. However, conflicting results have been reported regarding the association between GPX8 expression level and colorectal cancer.^[12]^ This discrepancy may be due to the fact that previous studies did not differentiate between colon and rectal cancer. Our study found that GPX8 was highly expressed in COAD tissues, but not in READ tissues. Moreover, we included a larger sample size of 50 COAD patients in our study. Therefore, our findings suggest that GPX8 may serve as a potential therapeutic target and prognostic marker for COAD. Nonetheless, further studies are warranted to fully elucidate the role of GPX8 in cancer.

The precise underlying mechanism by which GPX8 impacts patient survival in COAD and STAD is not yet fully elucidated. However, several studies have proposed potential mechanisms. One such mechanism involves induction of GPX8 expression by hypoxia-inducible factors, which reduces proliferative signaling during hypoxia and/or receptor tyrosine kinases signaling, both of which contribute to oncogenesis.^[12]^ Another study by Zhang et al^[17]^ suggests that GPX8 is positively associated with angiogenesis, epithelial–mesenchymal transition, hedgehog signaling, IL6-JAK-STAT3 signaling pathway, inflammatory response, and KRAS signaling pathway, and it interacts with proteins focused on oxidative stress and reactive oxygen species. Further research is needed to fully understand the role of GPX8 in cancer and its potential as a therapeutic target or prognostic marker.

Immunotherapy plays a vital role in the clinical management of tumors. Our investigation reveals that GPX8 has differential effects on immune infiltration in COAD and STAD. In COAD, patients with low CD4 + T cell infiltration levels had higher overall survival rates, whereas in STAD, patients with low neutrophil infiltration levels had higher overall survival rates. Li et al^[18]^ reported that high GPX8 expression is correlated with increased infiltration of CD4 T cells and neutrophils, which may contribute to tumor progression. Similarly, Xu et al^[19]^ demonstrated that high GPX8 expression is associated with poor prognosis of lung adenocarcinoma and is related to the level of T cell infiltration. These findings suggest that GPX8 may inhibit tumor immune response by recruiting different immune cell infiltration in COAD and STAD. Further studies are required to elucidate the precise mechanisms underlying the role of GPX8 in tumor immune infiltration.

This study has several advantages and limitations. On the positive side, it sheds light on the role of GPX8 in predicting survival and immune infiltration in patients with colorectal and STAD. The study analyzed a cohort of patients, including GPX8 expression, immune infiltration, and survival rates, providing important insights into the potential mechanisms of GPX8 in regulating the tumor microenvironment and immune response. This study also provides a potential avenue for developing targeted therapies for colorectal and STAD. However, the study is retrospective in nature and cannot establish causality between GPX8 expression, immune infiltration, and survival rates. Furthermore, the validation part of this study relies on data from a single institution, which may not be representative of the broader patient population. The study also did not investigate the underlying mechanisms by which GPX8 affects immune infiltration and survival rates in colorectal and STAD.

## 5. Conclusion

In summary, the study highlights the potential role of GPX8 in predicting survival and immune infiltration in colorectal and STAD. While the findings suggest GPX8 may regulate the tumor microenvironment and immune response, the retrospective nature of the study and reliance on data from a single institution limit the generalizability of the findings. Nonetheless, the study provides important insights into the biology of these cancers and identifies GPX8 as a potential therapeutic target for future research. Further studies are needed to explore the underlying mechanisms of GPX8 and its potential as a therapeutic target for these cancers.

## Acknowledgments

The authors express their gratitude to all the patients and their families who participated in this research.

## Author contributions

**Conceptualization:** Ya-Wen Zou.

**Data curation:** Hai-Tao Wang.

**Formal analysis:** Yun Cheng.

**Investigation:** Zhi-Lin Liu.

**Supervision:** Ya-Wen Zou.

**Validation:** Ya-Wen Zou.

**Visualization:** Yun Cheng, Zhi-Lin Liu.

**Writing – original draft:** Ya-Wen Zou.

**Writing – review & editing:** Yun Cheng, Zhi-Lin Liu, Hai-Tao Wang.
